# Outcomes of patients with loco-regionally recurrent or new primary squamous cell carcinomas of the head and neck treated with curative intent reirradiation at Mayo Clinic

**DOI:** 10.1186/s13014-016-0630-x

**Published:** 2016-04-09

**Authors:** Kelly K. Curtis, Helen J. Ross, Ashley L. Garrett, Theresa A. Jizba, Ajay B. Patel, Samir H. Patel, William W. Wong, Michele Y. Halyard, Stephen J. Ko, Heidi E. Kosiorek, Robert L. Foote

**Affiliations:** Department of Internal Medicine, Division of Hematology/Medical Oncology, Mayo Clinic, 13400 East Shea Blvd., Scottsdale, AZ 85259 USA; Department of Radiation Oncology, Mayo Clinic, Jacksonville, FL USA; Department of Radiation Oncology, Mayo Clinic, Scottsdale, AZ USA; Department of Biomedical Statistics, Mayo Clinic, Scottsdale, AZ USA; Department of Radiation Oncology, Mayo Clinic, Rochester, MN USA

**Keywords:** Reirradiation, Recurrent squamous cell carcinoma head and neck, Survival outcomes, Toxicity

## Abstract

**Background:**

We reviewed outcomes of patients with loco-regionally recurrent (LRR) or new primary (NP) squamous cell carcinoma of the head and neck (SCCHN) treated at our institution with reirradiation (RRT).

**Methods:**

Patients received definitive RRT (DRRT) or post-operative RRT following salvage surgery (PRRT) from 2003 to 2011. Measured survival outcomes included loco-regional relapse free survival (LRFS) and overall survival (OS).

**Results:**

Among 81 patients (PRRT, 42; DRRT, 39), median PRRT and DRRT doses were 60 Gy (12–70 Gy) and 69.6 Gy (48–76.8 Gy). The majority of patients received IMRT-based RRT (*n* = 77, 95 %). With median follow-up of 78.1 months (95 % CI, 56–96.8 months), 2-year OS was 53 % with PRRT and 48 % with DRRT (*p* = 0.12); 23 % of patients were alive at last follow-up. LRFS at 2 years was 60 %, and did not differ significantly between PRRT and DRRT groups. A trend toward inferior LRFS was noted among patients receiving chemotherapy with RRT versus RRT alone (*p* = 0.06). Late serious toxicities were uncommon, including osteoradionecrosis (2 patients) and carotid artery bleeding (1 patient, non-fatal).

**Conclusions:**

OS of PRRT- and DRRT-treated patients in this series appears superior to the published literature. We used IMRT for the majority of patients, in contrast to several series and trials previously reported, which may account in part for this difference. Future studies should seek to improve outcomes among patients with LRR/NP SCCHN via alternative therapeutic modalities such as proton radiotherapy and by incorporating novel systemic agents.

## Background

Although 5-year survival rates for oral cavity and pharynx cancer have improved significantly since the 1970s (53 % 1975–1977 versus 66 % 2004–2010, *p* < 0.05) [1], this improvement likely is due to an increase in favorable-prognosis Human Papilloma Virus-associated oropharynx cancers rather than the development of more effective treatment. In contrast, the five year survival rate for laryngeal cancer has not improved since the 1970s. A substantial percentage of patients with loco-regionally advanced squamous cell carcinomas of the head and neck (SCCHN) continue to experience disease recurrence after upfront surgery and/or radiation therapy. An estimated 17–33 % of loco-regionally advanced SCCHN patients develop loco-regional recurrences following definitive radiotherapy, and new SCCHN primaries in previously radiated volumes may develop at a rate of 1 % per year [2–4].

Curative-intent treatment options for patients with loco-regionally recurrent or new primary SCCHN (LRR/NP SCCHN) include surgical salvage alone, surgery followed by radiotherapy with or without chemotherapy, or radiotherapy with or without chemotherapy for non-surgical candidates. Experience with reirradiation (RRT) for LRR/NP SCCHN has been reported by several groups and evaluated in prospective trials. RRT confers increased risk of serious side effects, including but not limited to myelopathy, osteoradionecrosis of the mandible (ORN), tracheo-esophageal fistulae and fatal carotid artery hemorrhage. Despite these risks, most studies show that RRT cures a modest percentage of LRR/NP SCCHN patients with acceptable rates of serious toxicities [5].

To better understand the outcomes of our LRR/NP SCCHN patients treated at Mayo Clinic with RRT, we conducted a retrospective review. We sought to understand what disease and treatment-related factors may be associated with survival outcomes in this patient population. We included only patients treated in the contemporary era, when intensity modulated radiation therapy (IMRT) was used for most patients.

## Methods

We retrospectively reviewed the medical records of LRR/NP SCCHN patients treated with curative-intent definitive (DRRT) or post-operative reirradiation (PRRT) with or without chemotherapy from January 1, 2003 through December 31, 2011. All patients were treated at Mayo Clinic sites in Minnesota, Florida or Arizona. The review was approved by the Mayo Clinic Institutional Review Board.

Primary aims were to determine patient characteristics, doses and techniques of radiotherapy used, the number of patients given chemotherapy and type of chemotherapy given, and to calculate survival outcomes including loco-regional relapse free survival (LRFS), distant metastasis free survival (DMFS), and the overall survival (OS) of LRR/NP SCCHN patients treated with RRT at our institution over this time period. We included only patients with a histologically confirmed diagnosis of LRR/NP SCCHN and who had undergone prior curative intent radiation (+/− surgery and/or chemotherapy). Patients with new primary non-squamous cell cancers of the head and neck undergoing RRT and those treated with RRT at centers outside of Mayo Clinic were not included. We did not include patients treated with RRT for palliative purposes.

Patients selected for RRT at Mayo Clinic underwent clinical and radiologic staging, with distant metastases excluding patients from consideration of curative-intent RRT. All patients had localized disease, and previous radiation records were carefully reviewed. Patients were more than 12 months from prior course of radiation therapy. All patients underwent CT-based treatment planning with thermoplastic mask immobilization. The majority of patients were treated with IMRT. Minimum target volume included gross disease plus margin in the DRRT patients. Cumulative spinal cord doses were kept below 60 Gy.

Statistical analyses were performed using JMP version 11. Demographic and treatment data were tabulated by treatment group (PRRT versus DRRT), with differences between subgroups defined by categorical factors determined using the chi-squared test of independence and one way analysis of variance for subgroups defined by continuous variables. We used Kaplan-Meier methods to calculate median follow-up time from diagnosis of LRR/NP SCCHN, median time from date of first primary SCCHN to LRR/NP SCCCHN, median OS for the entire group and by treatment method (PRRT verus DRRT), and median LRFS and DMFS. LRFS and DMFS were measured from the date of completing RRT to date of diagnosis of loco-regional recurrence or distant metastases. OS was measured from date of diagnosis of LRR/NP SCCHN to date of death or last follow-up. Patients were considered censored at last follow-up date if the patient did not experience the event of interest (local-regional relapse, distant relapse or death). Cox proportional hazards model was used to determine hazard ratios for LRFS, DMFS and OS by subgroups as follows: LRR versus NP SCCHN, age < or ≥ 60 years at diagnosis of LRR/NP SCCHN, sex, tobacco abuse (< or ≥ 20 pack-years), PRRT versus DRRT, time since primary SCCHN (< or ≥ 24 months), use of chemotherapy with RRT, use of platinum versus cetuximab with RRT, and use of IMRT versus other technique with RRT. A *p* value less than 0.05 was considered significant.

## Results

We identified 81 patients (61 male, 20 female); 59 patients had LRR; 22 NP. Treatment included salvage surgery with PRRT (42 patients); 39 patients received DRRT. Table 1 shows patient demographics and other disease-related data from the cohort. There were no significant differences between PRRT- and DRRT-treated patients with respect to original primary site, recurrence site or new primary site. Human Papilloma Virus (HPV) status was not routinely tested, and therefore we were unable to perform any subgroup analyses based on this prognostic factor.

**Table 1:** Demographics and disease-related information by treatment group among 81 LRR/NP SCCHN patients undergoing reirradiation at Mayo Clinic

Characteristic	Surgery/PRRT n (%)	DRRT n (%)	*P*
# patients	42 (52)	39 (48)	
Sex			
Male	34 (81)	27 (69)	0.22
Female	8 (19)	12 (31)	
Age, years			
Median (range)	61 (34–83)	65 (36–83)	0.23
Original primary site			
BOT/OP	10 (24)	14 (36)	0.23
OC/FOM	7 (17)	3 (8)	
Larynx	15 (36)	17 (44)	
Sinus	2 (5)	0 (0)	
Other	4 (9)	4 (10)	
Unknown primary	4 (9)	1 (2)	
Relapse at primary site	17 (41)	11 (28)	
BOT/OP	2 (12)	5 (46)	0.20
OC/FOM	3 (18)	2 (18)	
Larynx/stoma	10 (58)	4 (36)	
Sinus	1 (6)	0 (0)	
Nasal cavity	1 (6)	0 (0)	
Relapse at other site, not considered NP	14 (33)	17 (44)	
BOT/OP	0 (0)	1 (6)	0.06
OC/FOM	1 (7)	0 (0)	
Sinus	1 (7)	0 (0)	
Nasopharynx	1 (7)	1 (6)	
Contra. Neck	7 (50)	2 (11)	
Ipsilat. Neck	4 (29)	10 (59)	
Supraclavicular	0 (0)	1 (6)	
Trachea	0 (0)	2 (11)	
NP site	11 (26)	11 (28)	
BOT/OP	2 (18)	7 (64)	0.07
OC/FOM	2 (18)	0 (0)	
Larynx	5 (45)	2 (18)	
Sinus	1 (9)	0 (0)	
Nasal cavity	1 (9)	0 (0)	
Nasopharynx	0 (0)	2 (18)	
Mean LRR/NP size, cm (SD)	3.4 (1.48)	3.2 (0.98)	0.785
Median # positive lymph nodes (range)	1 (0–9)	N/A	
LRR/NP Grade			
2	6 (16)	2 (9)	0.19
3	20 (56)	18 (78)	
4	10 (28)	3 (13)	
# ECE	19 (45)	N/A	
# LVI	10 (24)	N/A	
# perineural invasion	10 (24)	N/A	
# positive margins	11 (26)	N/A	
Tobacco use			
0	6 (14)	8 (21)	0.77
<20 PY	6 (14)	5 (13)	
≥20 OY	25 (60)	22 (56)	
UTQ	5 (12)	4 (10)	
Alcohol abuse by history	14 (33)	10 (26)	0.41

*BOT* base of tongue, *DRRT* definitive reirradiation therapy, *ECE* extracapsular extension, *FOM* floor of mouth, *LVI* lymphovascular invasion, *LRR* loco-regionally recurrent, *NP* new primary, *OC* oral cavity, *OP* oropharynx, *PRRT* post-operative reirradiation therapy, *PY* pack-year, *SD* standard deviation, *UTQ* unable to quantify

Table 2 provides details about concurrent chemotherapy with RRT and the radiotherapy techniques used both for primary radiotherapy (where such information was available in the medical record) and RRT. The median total dose of prior radiation for the cohort was 66 Gy (26.4–79.2 Gy). Use of chemotherapy with the first course of radiation could not be determined from the records of 57 patients. In the RRT setting, almost all DRRT-treated patients received 70 Gy or more, whereas most PRRT-treated patients received at least 60 Gy. A majority of patients in both DRRT- and PRRT-treated groups received concurrent chemotherapy. Cisplatin as a radiosensitizing agent was given to more PRRT patients than DRRT patients, for whom a majority received either cetuximab or carboplatin. In the PRRT group, more patients with positive margins received chemotherapy (9/11, 82 %) than patients with negative margins (11/21, 47.6 %, *p* = 0.05). Of patients with 1 or more lymph nodes involved, 21 (84 %) received chemotherapy.

**Table 2:** Chemotherapy and radiotherapy data for LRR/NP SCCHN patients undergoing reirradiation at Mayo Clinic

	Surgery/PRRT	DRRT	*P*
RRT + concurrent chemotherapy	28 (67)	32 (82)	0.11
Type			
Cisplatin	17 (61)	7 (22)	0.002
Cetuximab	8 (29)	11 (34)	
Cisplatin + cetuximab	0	3 (9)	
Carboplatin	1 (3)	5 (16)	
Other	2 (7)	6 (19)	
Median primary radiotherapy dose	66 Gy (50–72 Gy)	66 Gy (26.4–79.2)	0.62
Median RRT dose	60 Gy (12–70 Gy)	69.6 Gy (48–76.8 Gy)	<0.0001
RRT dose			
< 60 Gy	11 (26)	1 (3)	-
≥ 60 to < 70 Gy	25 (60)	8 (20)	
≥ 70 Gy	6 (14)	30 (77)	
Primary radiotherapy technique			
IMRT	22 (52)	11 (28)	0.95
3D conformal	13 (31)	18 (46)	
No information	7 (17)	10 (26)	
RRT technique			
IMRT	36 (86)	38 (97)	0.08
IMRT/IORT	3 (7)	0	
3D conformal	3 (7)	1 (3)x	

*DRRT* definitive reirradiation therapy, *IMRT* intensity modulated radiation therapy, *IORT* intra-operative radiation therapy, *PRRT* post-operative reirradiation therapy, *RRT* reirradiation

The median follow-up time for the entire cohort from date of LRR/NP diagnosis was 78.1 months (95 % CI, 56–96.8 months). Five patients were lost-to-follow-up (not seen for ≥ 2 years at time of analysis with no recorded date of death). Median time from date of first primary SCCHN to LRR/NP SCCHN was 33.2 months (95 % CI, 25–48.6 months). No significant difference in time from date of first primary SCCHN to LRR/NP SCCHN was found between the PRRT and DRRT patients (median time 28.2 months, 95 % CI, 20–48.6 months for PRRT, versus 38.2 months, 95 % CI, 25.1–78.2 months for DRRT, *p* = 0.99).

**Fig. 1:**
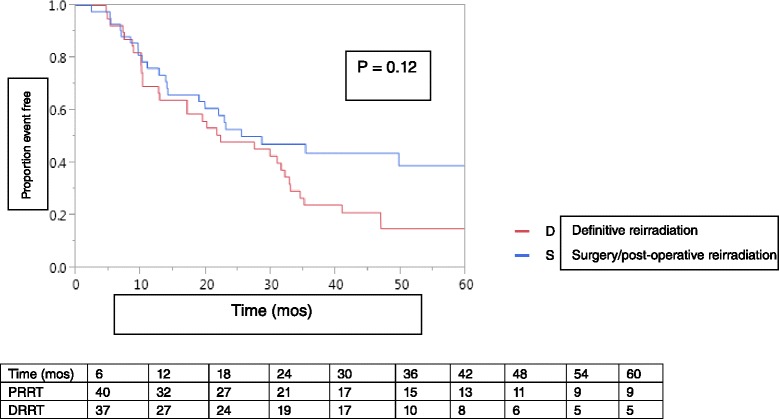
Kaplan-Meier plot of overall survival among LRR/NP SCCHN patients treated with DRRT versus PRRT

OS at 2 years for the entire cohort was 50 %, with a median OS of 22.2 months (95 % CI, 17–29.8 months). We found no difference in OS between PRRT patients (53 % at 2 years, median OS 28.6 months, 95 % CI, 14.1–61.6 months) versus DRRT patients (48 % at 2 years, median OS 22.2 months, 95 % CI, 12.7–32.1 months, *p* = 0.12, Fig. 1). Neither concurrent chemotherapy nor RRT technique affected OS on univariate analysis (Table 3). Total RRT dose (≥70 Gy versus < 70 Gy) did not impact OS of the DRRT-treated group. However, a trend toward inferior OS among PRRT patients who did not receive at least 60 Gy compared to PRRT-treated patients receiving at least 60 Gy was observed (10.2 months, 95 % CI, 2.3–84.1 months versus 35.3 months, 95 % CI, 19.8–61.6 months, *p* = 0.08).

**Table 3:** Univariate analyses of survival outcomes by patient and treatment-related factors

	Overall Survival, HR (95 % CI)	Time to loco-regional recurrence, mos (95 % CI)	Time to distant metastases, mos (95 % CI)
LRR vs. NP SCCHN	0.84 (0.49–1.49)	1.05 (0.51–2.38)	0.99 (0.58–1.77)
	*P* = 0.53	*P* = 0.91	*P* = 0.96
Age at diagnosis of LRR/NP SCCHN	1.4 (0.84–2.36)	1.01 (0.52–2.03)	1.62 (0.97–2.77)
< 60 vs. ≥ 60	*P* = 0.2	*P* = 0.97	*P* = 0.07
Sex	0.92 (0.52–1.71)	0.53 (0.27–1.15)	0.98 (0.54–1.91)
Male vs. Female	*P* = 0.78	*P* = 0.1	*P* = 0.95
Tobacco abuse	0.67 (0.86–2.72)	1.84 (0.26–1.12)	0.82 (0.71–2.16)
< 20 PY vs. > 20 PY	*P* = 0.16	*P* = 0.1	*P* = 0.48
Post-operative vs. Definitive RRT	0.67 (0.4–1.11)	0.62 (0.31–1.23)	0.67 (0.4–1.1)
	*P* = 0.12	*P* = 0.17	*P* = 0.12
Time since primary SCCHN	0.77 (0.44–1.3)	1.19 (0.43–1.73)	0.79 (0.75–2.18)
< 24 mos vs. ≥ 24 mos	*P* = 0.33	*P* = 0.63	*P* = 0.39
Chemotherapy vs. no chemotherapy with RRT	1.47 (0.82–2.84)	2.43 (1.02–7.16)	1.12 (0.66–1.98)
	*P* = 0.2	*P* = 0.04	*P* = 0.68
Platinum vs. cetuximab with RRT	0.65 (0.36–1.22)	0.4 (0.19–0.86)	0.65 (0.33–1.31)
	*P* = 0.18	*P* = 0.02	*P* = 0.22
IMRT versus other	0.99 (0.4–3.32)	2.03 (0.43–36.2)	1.21 (0.49–4.04)
	*P* = 0.99	*P* = 0.44	*P* = 0.71

*CI* confidence interval, HR hazard ratio, IMRT intensity modulated radiation therapy, LRR loco-regionally recurrent, NP new primary, NR not reached, PY pack-years, RRT reirradiation therapy, SCCHN squamous cell carcinoma of head and neck

**Fig. 2:**
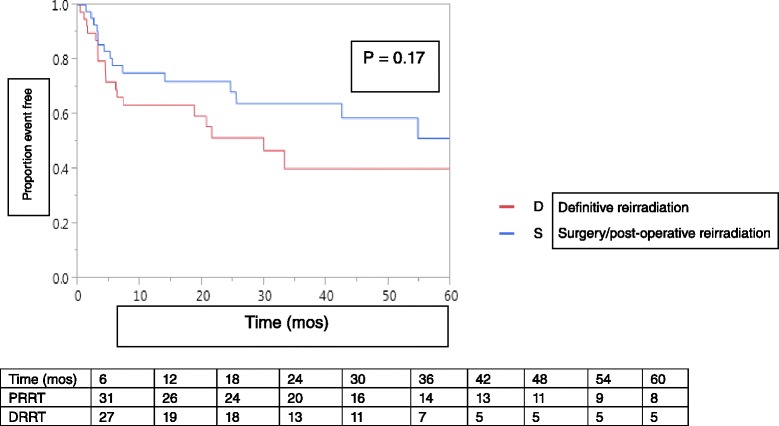
Kaplan-Meier plot of loco-regional relapse free survival among LRR/NP SCCHN patients treated with PRRT or DRRT at Mayo Clinic

LRFS at 2 years for the entire cohort was 60 %, with a median LRFS following RRT of 54.7 months (95 % CI, 24.5 months – NR). Loco-regional recurrences following RRT occurred in 34 patients (42 %), 28 (82 %) of which occurred within 2 years. Figure 2 shows no significant difference in loco-regional recurrence free survival according to PRRT or DRRT treatment groups. Overall, most loco-regional recurrences were within the RRT treatment volume (*n* = 21, 62 %). By treatment group, 19 DRRT patients developed loco-regional recurrence, 13 (68 %) of which were in-field, and 15 PRRT patients developed loco-regional recurrence, 8 (53 %) of which were in-field (*p* = 0.37 for in-field recurrences with DRRT versus PRRT). Use of IMRT with RRT did not impact time to loco-regional recurrence.

DMFS at 2 years for the entire cohort was 53 %, with a median DMFS following RRT of 27.4 months (95 % CI, 18.9–34.5 months). No significant difference in DMFS between PRRT and DRRT-treated patients was observed (median 32 months, 95 % CI, 19.7–49.1 months with PRRT versus 21.6 months, 95 % CI, 10.2–32.8 months with DRRT, *p* = 0.11). Distant metastases developed in 18 patients (22 %), with 15 of these 18 patients (83 %) developing distant metastases within 2 years of completing RRT. Concurrent chemotherapy had no impact on DMFS on univariate analysis (Table 3).

Univariate analysis revealed no statistically significant differences in LRFS, DMFS or OS based on any of the variables analyzed except for receipt of platinum chemotherapy with RRT and concurrent chemotherapy/RRT, both associated significantly with LRFS (Table 3). Concurrent chemotherapy with RRT was associated with inferior LRFS, whereas platinum chemotherapy versus cetuximab with RRT was associated with improved LRFS.

Acute toxicity mostly consisted of radiation dermatitis and mucositis (71 of 81 patients, 88 %). Gastrointestinal toxicities (nausea, vomiting and/or diarrhea) were reported in 13 patients (16 %). Among surviving patients, 2 cases of ORN (10 %) and 1 non-fatal carotid artery bleeding aneurysm, successfully treated with embolization (5 %), were reported, with no reported spinal cord injuries. Long term toxicity data are further summarized in Table 4. Late toxicity was incompletely documented in the medical record and could not be reported in this analysis.

**Table 4:** Long term complications reported among LRR/NP SCCHN patients undergoing reirradiation at Mayo Clinic alive at last follow-up, by treatment modality

	Surgery/PRRT	DRRT
	*n* (%)	*n* (%)
Carotid artery aneurysm	1 (2)	0
Dysphagia	2 (5)	0
Esophageal stenosis	1 (2)	1 (3)
Feeding tube at last f/u	1 (2)	2 (5)
Hypothyroid	1 (2)	0
Impaired shoulder ROM	2 (5)	0
Ischemic retinopathy	1 (2)	0
None reported	5 (12)	1 (3)
Osteoradionecrosis	1 (2)	1 (3)
Soft tissue fibrosis	1 (2)	0
Tracheoesophageal fistula	1 (2)	0
Xerostomia	2 (5)	2 (5)

*ROM* range of motion

## Discussion

Patients previously treated for SCCHN are at risk for new primary cancers in the treatment volume due to “field cancerization” effect, and most cases of LRR/NP SCCHN occur in or near areas previously treated with surgery and/or radiotherapy [5]. LRR/NP SCCHN therefore is less amenable to cure with surgery and/or RRT, owing to radiation resistance mechanisms in the new or recurrent cancer as well as the local morbidity imposed by re-treatment [5]. Here we report the outcomes of LRR/NP SCCHN patients treated with RRT at Mayo Clinic between 2003 and 2011. The long term outcomes of our patients are similar if not superior to previously reported findings from other groups. Our analysis therefore confirms several key previously published findings.

First, our results confirm that RRT can achieve long-term disease control in a modest percentage of patients with LRR/NP SCCHN. With a median follow-up of approximately 6.5 years, 23 % of patients in our series were alive at last follow-up; 50 % were alive at 2 years. These data support the use of RRT for LRR/NP SCCHN patients as a salvage treatment approach despite its known risk of side effects.

Outcomes of patients treated with either PRRT or DRRT in our series appear superior to those from previous reports. The 2-year OS for PRRT-treated patients we observed is higher than the 45 % 2-year OS observed in the prospective phase III study of salvage surgery with or without post-operative RRT with chemotherapy conducted by the Groupe d’Etude des Tumeurs de la Tête Et du Cou and Groupe d’Oncologie Radiothérapie Tête et Cou (GORTEC) groups [6]. Our 2-year OS for DRRT-treated patients (48 %) is better than that observed in the prospective RTOG 9610 trial of 4 weekly cycles of 5-fluorouracil/hydroxyurea with 60 Gy at 1.5 Gy in twice-daily fractions, which demonstrated 2-year OS of 15.2 % (95 % CI, 7.3–23.1 %) [7]. The 2-year OS with DRRT reported here also is better than was found in the prospective RTOG 9911 trial of cisplatin plus paclitaxel IV daily times 5 days, every 2 weeks, for 4 courses with split-course RRT, 60 Gy total dose, 1.5 Gy BID times 5 days, every 2 weeks, times 4 courses. That study demonstrated a 2-year OS of 25.9 % (95 % CI, 17.3–35.3 %), and also found inferior median OS for those treated more than 36 months after first radiation (10.8 months) versus 14.1 months if treated within 36 months of first radiation, a finding which we could not confirm [8]. Both RTOG studies used radiation treatment regimens other than conventional fractionation and RTOG 9610 did not use IMRT, which was used for the majority of patients in our study, and might contribute to the difference in OS we observed. IMRT has been shown to improve LRFS and OS in the primary treatment of SCCHN and in LRR/NP SCCHN [9, 10]. Lee and others [9] found that among patients treated with RRT using conventional techniques (31 patients) versus IMRT (74 patients) between 1996 and 2005, LRFS was 52 % at 2 years with IMRT versus 20 % with conventional RRT (*p* < 0.001; on univariate analysis, OS was superior with IMRT (HR for death 0.57, 95 % CI, 0-35-0.93, *p* = 0.03). We could not confirm this finding on univariate analysis, although the small numbers of patients treated with RRT technique other than IMRT in our series probably limits our ability to detect a survival difference by technique. The demographics of our patient population mirrored the study population of the RTOG 9610 and RTOG 9911 studies in terms of age (median 62 and 60 years, respectively), gender (over 75 % male in both studies) and time since primary radiation (median 2.5 years and 39.6 months, respectively). Therefore, the differences in outcomes we observed are not explained by differences in these factors.

The 2-year OS of our DRRT-treated patients also appears superior to outcomes of DRRT-treated patients reported in many studies published since 2000 [7, 8, 11–27]. In these studies, OS at 2-years of 20 % or less was reported in 9 of 19 series; only 1 report [22] found a 2 year OS similar to the Mayo Clinic experience (45.1 %). In one of the largest series of DRRT outcomes reported to date, De Crevoisier and others reported a 2-year OS of 21 % (95 % CI, 15–29 %) [28]. We used a higher median radiation dose than was given in most of the series reporting 2-year OS ≤ 20 %, where radiation doses ranged from 34 to 60 Gy (8 of 9 studies). Higher RRT doses may be required to achieve long term disease control, a hypothesis supported by a previous retrospective analysis of 103 patients undergoing RRT, in which RRT dose > 50 Gy was associated with improved survival [29]. Our finding that PRRT-treated patients treated with at least 60 Gy of RRT had a trend toward improved OS compared to those treated with lower PRRT doses also lends support to this hypothesis. However, published evidence to the contrary exists, as a prospective phase I study by Seiwert et al. found 2-year OS of just 17.2 % despite a median RRT dose of 70 Gy [21]. In the current era, PET/CT staging routinely is used to better select appropriate patients for RRT, thereby potentially favorably impacting the outcomes we report in comparison to older series.

Surgical salvage is believed to be the most effective treatment for LRR SCCHN, a conclusion based on a meta-analysis of 32 published reports of salvage surgery in which 5 year OS of 39 % was reported [30]. Although patients in our study treated with surgical salvage and PRRT did not have a significantly different OS from DRRT-treated patients, we did observe a trend toward improved OS among PRRT patients. The 2-year OS of PRRT patients in our report (53 %) mirrors that found in multiple previous reports, where OS at 2 years ranges from 21 to 81 % [11, 12, 31–37]. This finding probably is explained by selection bias: patients selected for salvage surgery generally are in better overall health and have smaller tumors, potentially creating a selection bias in favor of PRRT compared to DRRT. Greater use of cetuximab than cisplatin with DRRT as observed in our series also suggests selection bias of patients with more comorbidities and/or poorer functional status for DRRT in lieu of surgery/PRRT, potentially skewing the survival outcomes of DRRT-treated patients.

We found no significant impact on survival outcomes with the addition of concurrent systemic chemotherapy to RRT. A large percentage of LRR/NP SCCHN patients in our cohort received systemic therapy, typically cisplatin with PRRT and cetuximab with DRRT, but the choice of platinum versus cetuximab did not impact OS on univariate analysis. Receipt of any form of chemotherapy was associated with inferior LRFS. We suspect this finding also relates to a selection bias, wherein patients with higher risk, more advanced LRR/NP SCCHN were chosen for “aggressive” treatment with chemotherapy and RRT. Indeed, a large percentage of patients with positive surgical margins received chemotherapy, and the majority of patients treated with concurrent chemotherapy also had nodal involvement. Therefore, the inferior LRFS observed with chemotherapy plus RRT compared to RRT alone most likely was due to disease related factors, rather than a deleterious effect of chemotherapy on RRT. Given that randomized studies of chemotherapy with radiation for newly diagnosed, previously untreated SCCHN show a survival benefit of approximately 6 % [38], it is not surprising that no OS difference was found between patients treated with RRT and chemotherapy versus RRT alone in this retrospective series with a smaller number of patients.

The incidence of long-term, serious adverse effects from RRT in our study was low. Inadequate documentation and patients lost to long term follow-up very likely lead to an underestimation of the late side effects of RRT. Of those patients alive at last follow-up, few serious adverse events had been recorded, but these may be underestimated due to the lack of systematic documentation of adverse events as would have occurred had this been a prospective trial. The vast majority of toxicities were limited in duration to the period of RRT and subsequently resolved. In contrast, prospective trials of RRT have shown higher rates of late grade 3–4 toxicity. RTOG 9610 reported 19.4 % late (>90 days) grade 3 and 3 % late grade 4 toxicity, mostly pharyngeal and esophageal, including 2 fatal hemorrhages [7]. RTOG 9911 reported approximately 17 % of patients having grade 3 and 17 % grade 4 late toxicity, with 3 of 8 treatment related deaths due to carotid artery hemorrhage [8]. Another prospective phase I/II trial of low dose daily cisplatin/paclitaxel with split course RRT reported 34 % of patients with long term toxicities including 2 each of ORN, carotid artery hemorrhage, trismus and fistula formation [18]. Late grade 3 toxicity rates as high as 47.5 % have been reported in retrospective series, some of which did not use IMRT [29]. A pooled analysis found a rate of carotid artery rupture of 2.6 % among 1554 patients in 27 published articles, with 76 % of events being fatal [39]. Conventional or hyperfractionated techniques as opposed to accelerated hyperfractionation, and use of IMRT versus 3D-conformal RT may reduce serious long term side effects of RRT [39, 40]. The low rates of serious long term adverse effects from RRT we report could be due to the use of a hyperfractionated schedule with IMRT in the majority of patients, but again it would be difficult to make firm conclusions given the small sample size and high chance of underreporting of toxicities.

Stereotactic body radiotherapy (SBRT) has gained attention in recent years as a viable alternative to conventional RT for patients with LRR/NP SCCHN. In comparison to conventional RT, which treats larger volumes over more elapsed days using conventional dose fractionation, SBRT treats smaller volumes, better spares normal tissues, delivers greater biological dose, and is given over 5 or fewer elapsed treatment days. Disadvantages of SBRT include more complex planning and higher doses per treatment to previously irradiated tissues, potentially increasing toxicity depending on the tissue type. Several groups have published their experience with SBRT for LRR/NP SCCHN. In these series, median RT doses have ranged from 24 to 48 Gy. Overall response rates ranging from 58.1 to 81 % and median OS of 6.7 to 16.2 months have been reported. Up to 17.8 % rates of grade 3 toxicity, including fistula and carotid artery hemorrhage have been observed [41]. A recent prospective phase II study of cetuximab with SBRT (consisting of 40–44 Gy in 5 fractions on alternating days over 1–2 weeks) showed a 51 % complete and partial response rate and 14 % with stable disease. Median OS was 10 months (95 % CI, 7–16 months), and 37 % were free of loco-regional progression at 1 year (95 % CI, 23–53 %). Observed toxicity included a 6 % rate of late grade 3 toxicity (2 patients with fistula, 1 patient with dysphagia, 1 patient with an arterial bleed, but no incidence of carotid artery hemorrhage). Quality of life was stable or improved compared to baseline in 62 % of patients [42]. Early results of SBRT are encouraging, but prospective trials comparing outcomes with SBRT to 3D-conformal RT or IMRT are lacking. Several trials of SBRT for LRR/NP SCCHN are ongoing.

The outcomes of RRT for LRR/NP SCCHN in our study compare favorably to those reported in the literature. However, as with any retrospective review, there are several limitations to our study. The variety of LRR/NP sites among patients included in the series and the varying radiotherapy techniques and chemotherapy regimens used limit our ability to determine if RRT is more beneficial for some LRR/NP sites than others or to make firm conclusions about the optimal dose, volume and technique of RRT to use and which chemotherapy agent (if any) maximizes radiosensitization. The survival outcomes and toxicity rates we report may contain inaccuracies due to loss of patients to long term follow up, and our outcomes may differ from other reports owing to strict patient selection criteria and routine use of PET/CT for staging. Despite these limitations, the outcomes of patients with LRR/NP SCCHN treated with RRT at Mayo Clinic demonstrate that a subset of patients can achieve long-term survival with acceptable toxicities. Studies of molecular markers to guide treatment in this heterogeneous group of patients are desperately needed. The use of particle beam radiotherapy may improve sparing of normal tissues and allow radiation dose escalation. We plan to explore the role of proton beam radiotherapy for LRR/NP SCCHN; these data presented here will serve as a basis for comparison of outcomes with photon-based radiotherapy to proton radiotherapy in this patient population.

## Conclusions

In conclusion, patients with LRR/NP SCCHN require treatment to spare them from morbidity of their disease and to offer them the best hope of long term survival. Such patients should be offered surgical salvage when appropriate with or without RRT or DRRT for non-surgical candidates, in keeping with the American College of Radiology Appropriateness Criteria for retreatment of recurrent SCCHN [43]. Higher doses of radiation likely are required to produce long term disease control. The roles of treatment modalities such as SBRT and proton beam therapy need to be better defined by prospective trials. Ongoing research into the best radiation technique and most effective systemic chemotherapeutic agents will clarify treatment algorithms for LRR/NP SCCHN patients in coming years.
